# Understanding system interdependencies in sustainable paper production from residue grass biomass: Insights from fuzzy cognitive mapping

**DOI:** 10.1038/s41598-024-84358-4

**Published:** 2025-01-09

**Authors:** Zhengqiu Ding, Philipp Grundmann

**Affiliations:** 1 Innovations in Sociotechnical Systems, Department of Technology Assessment, Leibniz Institute for Agricultural Engineering and Bioeconomy (ATB), 14469 Potsdam, Germany; 2 Department of Agricultural Economics, Humboldt-Universität zu Berlin, 10117 Berlin, Germany

**Keywords:** Pulp and paper industry, Sustainable production, Grass biomass, Fuzzy cognitive maps, Stakeholders, Circular bioeconomy, Environmental social sciences, Energy and society, Socioeconomic scenarios, Sustainability

## Abstract

This research investigates the pulp and paper industry's transition to sustainability by valorizing unused roadside and natural grasses for paper production. Large-scale production from residual grass poses multifaceted challenges, requiring collaboration across stakeholders, from biomass collection to manufacturing. To understand key drivers and barriers within this complex system, experts from various fields, including local farmers, researchers, policymakers, and industry executives were interviewed, leading to the development of a Fuzzy Cognitive Map (FCM). The analysis explores various scenarios to assess how socio-economic, technological, and political factors influence the transition to low-carbon practices. These scenarios highlight the effects of varying levels of technology development, economic conditions, and policy support on the transition's progress and outcomes. Results show that the system is highly sensitive to shifts in socio-economic and political conditions. Political interventions play a crucial role, especially during energy crises and increased public demand for sustainable solutions. Grass-based paper production is seen as a viable pathway, but challenges such as the economic feasibility of emerging technologies remain. We recommend targeted policies to improve the economic viability of grass-based products and optimize biomass allocation between energy and bio-based products, ensuring a more balanced and sustainable transition.

## Introduction

The pulp and paper industry (PPI) faces a complex challenge of advancing towards a sustainable bioeconomy while integrating green technologies to ensure long-term growth^1^. Since the 2010s, the rise of e-commerce, further accelerated by COVID-19 lockdowns, has led to a significant increase in the demand for cardboard. By 2021, global production of printing & writing paper, paperboard, packaging paper, and household & sanity paper had reached 809 million metric tons^2^. Meeting these objectives call for innovative use of resources and the promotion of environmentally friendly solutions to transform this sector^3,4^. Recognizing emerging sustainability megatrends is key to shaping the future trajectory of the PPI^4^. Notably, this sector is one of the most carbon-intensive industries in the European Union (EU). High energy consumption is a key requirement for manufacturing processes in these industries^3,5,6^. Moreover, the depletion of forests resulting from wood extraction for wood-based bioeconomy has had significant environmental consequences^7^. This depletion increases the risks, costs, and constraints associated with conducting business and heightens competition. Nevertheless, it also presents new business opportunities to materialize alternative resources^3^.

Global pulp consumption for paper production highlights the dominance of virgin pulp, with approximately 196 million metric tons produced worldwide in 2021^2^. Wood pulp accounts for about 63% of this total, waste paper pulp represents 34% and non-wood pulp makes up the remaining 3%^8^. Increasing the efficient utilization of non-wood fiber resources, including grasses, holds significant potential in optimizing raw materials for papermaking processes and offers a promising solution to mitigate environmental destruction caused by deforestation^9^. Evidence has been found in the ecological footprints for the pulping from non-wood fiber compared with the harvested forestry^10^. The properties, such as strength, thickness, brightness, breaking length, demonstrated by paper products made from grass pulp affirm the suitability of grass as a viable alternative to wood for papermaking^11^. Furthermore, the substantial biomass output of non-wood plants, coupled with the abundance of agricultural residues, holds the potential to yield significant quantities of non-wood fibers. These fibers could serve as substitutes for a considerable portion of imported pulp^12^. Transitioning to non-wood fiber for paper production presents a multifaceted system change with many different implications from a sustainability perspective. It is essential to consider socio-economic factors, such as the direct and indirect effects of any land-use changes resulting from increased biomass demand^13^. These changes have also given rise to significant socio-ecological implications, for instance organizing society–nature relations^14^. One potential sustainable biomass yet underexplored is vegetation from roadside verges. These areas could serve as valuable biomass sources, leading to positive socio-ecological outcomes such as enhanced biodiversity and nutrient recycling^15^. Transforming these green biomass could significantly advance efforts toward a more resource-efficient and circular bioeconomy^16,17^. Non-woody biomass offers numerous economic benefits, providing farmers with additional income through the use of residues such as grasses^18^.

The existing literature predominantly highlights non-wood-based paper production concerning technology development and feasibility, with a focus on product properties and viable sources^8,19–22^. Despite that, studies on socio-economic barriers remains limited^3^. Specifically, there is a lack of research on stakeholders' perspectives regarding the use of roadside and nature grass for papermaking. Understanding these social dynamics and their complex interconnections with economic, technological, political, environmental, and industrial factors is crucial for promoting the adoption of non-wood-based paper production on a broader scale.

The approaches study social aspects of this industry include social life cycle assessment^23^, Delphi method^1^, and social network analysis^24^. Achieving transformative change for sustainability requires innovative approaches that acknowledge the complexity of local systems and integrate stakeholder perspectives. Fuzzy Cognitive Maps (FCMs) provide an effective means to address this challenge by supporting decision-making processes rooted in human reasoning. They offer a powerful tool for representing systems as networks of concepts and causal relationships, with the strength and nature of these connections expressed in fuzzy terms^25,26^. The FCM approach has been applied in various studies. These include formulating effective bioeconomy policies^27–29^, assessing social acceptante^30,31^. Other studies focus on analyzing bio-energy production systems^32^, managing biomass at the farm level^33^, planning for regional bioeconomy^34^ and water resource management^35^. Additionally, the implementation of technological approaches for biomass management has been explored^36^. We apply the Fuzzy Cognitive Maps (FCM) tool to explore the complexities of the sustainability transition in the pulp and paper industry. This approach allows us to examine the interconnections between various socio-economic, environmental, and technological factors and to develop policy scenarios that support informed decision-making for sustainable development.

This work significantly contributes to future discussions in the literature on opportunities for decarbonizing the paper and pulp industry^3^. It also contributes on sustainable transitions by emphasizing actor-oriented approaches that explicitly connect individual-level perspectives with system-level analysis in transition studies^37^. To the best of our knowledge, it is the first to apply FCMs to investigate stakeholders' perspectives on transitioning to circular bioeconomy to utilize roadside and natural grass for paper production. Consequently, the research questions in the paper are as follows: What are the primary barriers hindering the transition process towards utilizing roadside and nature grass for paper production, and which drivers and policy actions show the most promise in facilitating this transition? Additionally, how do socio-economic, political, and technological factors interconnect with each other in this process? The contribution lies in systematically developing and comparing various factors involved in transitioning to non-wood-based paper production. This research assists decision-makers in business, politics, and civil society by identifying barriers and pathways for the future deployment of PPI.

## Literature review and theoretical background

### Challenges in utilizing non-wood-based fibers in the pulp and paper industry

**Fig. 1:**
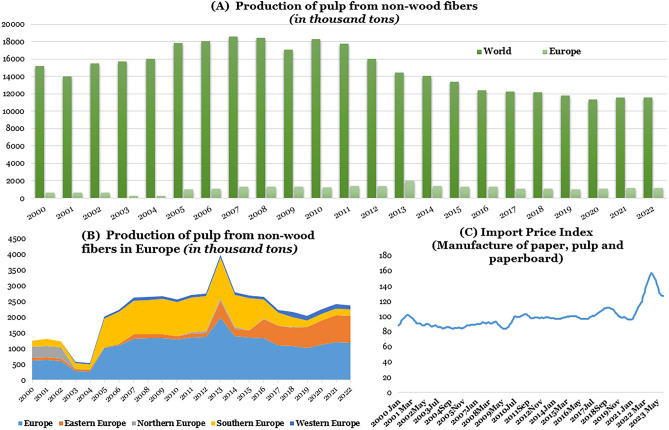
Pulp production from non-wood fiber and price index for paper, pulp, and paperboard manufacturing in Europe. (**A**) Comparison of non-wood based pulp production volumes between the world and Europe. (**B**) Breakdown of non-wood based pulp production across four European regions. (**C**) Trend in the price index for pulp in Europe.

The PPI has undergone significant transformations in response to environmental concerns and the urgency for sustainable practices. A notable development in this context is the exploration of alternative fiber sources, particularly grass-based fibers, which present a promising avenue for reducing the industry's environmental footprint^38^. As depicted in Fig. 1A and B, non-wood fiber production in Europe constitutes less than 10% of total wood production^2^. Upon closer examination of production across European regions, Eastern Europe dominates non-wood fiber production, while Northern countries contribute less, likely due to their abundant forestry resources. Western Europe, meanwhile, retains a relatively small share of overall production. On the other hand, as indicated in Fig. 1C based on the data from European Central Bank^39^, the import prices for manufacturing paper, pulp, and paperboard soared from 2020 to 2022. While there was a slight decrease in 2023, future projections indicate that prices will rise again and remain at a relatively high level. In this context, seeking alternative sustainable resources for paper production becomes increasingly relevant.

Despite notable progress in research and development using non-wood fiber for papermaking, critical gaps persist in the existing literature that need to be addressed. Foremost among these gaps is the absence of comprehensive studies on the variability of grass fibers sourced from different origins and its consequential effects on the quality and properties of paper products. Addressing this gap is necessary for ensuring consistent paper quality^20,21^. Although the environmental benefits of grass-based fibers are well-documented^10^, comprehensive life cycle assessments and economic analyses are still relatively scarce. A holistic understanding of grass-based paper production's environmental and economic sustainability is vital for informed decision-making^40,41^.

Research endeavors focusing on the development and optimization of processing technologies for grass-based fibers, encompassing pretreatment methods and pulping techniques, are essential for overcoming existing challenges in integration into the paper industry^8,19,22^. Of particular concern are the cost implications associated with the bulky nature of raw materials, which pose logistical challenges and may impede large-scale commercial operations^42,43^. Additionally, the PPI is undergoing significant changes, particularly in the development of new business models that integrate with the local bioeconomy and interface with socio-technical systems^3^. Related to this, a critical yet underexplored area pertains to market acceptance and consumer perceptions of paper products derived from grass fibers. Understanding the factors influencing consumer choices and preferences can drive adoption and inform market strategies^44,45^. The alignment of policies and regulations with the utilization of grass-based fibers in the paper industry represents a significant research gap. From a value chain perspective, addressing this gap requires coordinated policy efforts that integrate both top-down and bottom-up approaches. Such coordination is essential to developing a grass-based bioeconomy that balances the interests of various stakeholders across the value chain^44^. Additionally, the valorization of lignin offers a dual benefit: it not only enhances the economic value of biomass but also addresses critical issues related to waste management and environmental sustainability^46^. Investigating the impact of governmental policies, incentives, and regulations on the adoption and growth of grass-based paper production is crucial for creating a supportive environment for sustainable practices. Robust modeling tools are needed to understand and analyze these dynamics, capturing the nuances of these interconnections and predicting potential outcomes.

### Participatory modeling for regional bioeconomy development

Participatory modeling, rooted in post-normal science, recognizes the complexity, uncertainty, and value conflicts inherent in highly complex systems, advocating for collaborative approaches that actively involve diverse stakeholders to address urgent, real-world problems^47^. These problems are addressed within the studied system, through either the creation or assessment of models representing that system. When standard scientific methods fail to provide sufficient evidence, it becomes necessary to integrate local knowledge and engage in iterative participatory processes to formulate solutions that are both comprehensible and politically feasible while maintaining scientific rigor^48^. It is commonly recognized that stakeholders hold insights, perspectives, and expertise that can greatly enhance the research process in the bioeconomy^34,49^. In this context, the importance of various factors in sustainable bioeconomy development has been assessed. In the transition to a local bio-based energy production system, competitiveness, by-products, feedstock availability, and jobs were found to be the most important factors. Conversely, land availability, knowledge, existing symbiotic industry, and environmental sustainability were recognized to be the least important factors. Community acceptance and social capital consistently retained a moderate impact^34,50^. Interestingly, a contradictory finding emerges from another study examining the transition to bio-energy systems in different regions, where environmental factors were deemed most influential on decision-making processes related to low-carbon policies^51^. This highlights the complexity of transitioning to a bioeconomy, necessitating careful examination of each system. Notably, in the development of technology to valorize biomass, political factors emerge as the most influential, surpassing socio-technical factors in their impact^36^. This underscores the need for holistic and participatory analyses in navigating the complexities of transitioning to a sustainable bioeconomy.

## Materials and methods

### Study case

This study uses the case of grass fibers for paper production using grass biomass sourced from roadside areas and national parks in the Netherlands. This case is a demonstration action developed within the GO-GRASS project. In the Netherlands, grass sourced from roadside areas had historically served as cattle feed during the 1970s. However, by the 1980s, farmers' demand for such grass decreased, leading to a shift in disposal methods, with a growing portion being deposited at waste sites. Subsequently, from the late 1980s onward, composting emerged as the primary disposal method for roadside grass. Meanwhile, grass sourced from natural areas found utility in both cattle feed and stable litter on farms^52^. Over recent decades, a significant portion of these natural areas has been designated for conservation purposes. Even so, the nutritional value of grass from these lands has declined over time, attributed to reduced nutrient content and biomass removal practices aimed at nitrogen reduction.

Grass clippings from road verges contribute to approximately 30% of green waste generated from public green spaces, amounting to roughly 900 kilotons per year^53^. The majority of this grass is collected and composted, while the remaining portion is subjected to either fermentation or left to decompose onsite. Although composting presents a circular solution, it yields a low economic value, causes considerable amounts of CO_2_ emissions, and does not generate any revenues from the grass clippings to verge owners^53^. Consequently, both roadside and nature area managers are exploring alternative uses for grass resources^52^.

As illustrated in Table 1, the shift in the utilization of both roadside and natural grass resources, from cattle feed to waste disposal and the exploration of alternative uses reflects evolving agricultural practices as well as economic and ecological considerations. This transformation emphasizes the necessity for a comprehensive assessment of the economic and environmental impacts associated with the management of roadside and natural grass. Moreover, it presents an opportunity to explore new sustainable uses for these abundant grass resources in the Netherlands^54,55^. Considering the substantial volume of grass generated annually, examining the feasibility of utilizing grass biomass for the regional bioeconomy transition appears highly relevant.

**Table 1:** Overview of roadside and nature grass usage and estimated biomass quantities in the Netherlands.

	Roadside grass	Nature grass	References
Usage of the grass			
1970s	Cattle feed	Cattle feed and litter in stables	Vural Gursel et al.^52^
1980s	Demand for farmers decreased and often more deposited at waste dumps		
1980 onwards	Composting		
Recent years	Less attractive to farmers, and demanding alternative use of the grass and development for bioeconomy (e.g., bioenergy, paper )		Vural Gursel et al.^52^
Estimated quantity	900,000 tons year^-1^		Rijksdienst voor Ondernemend Nederland^53^
	225,000 ton DM year^−1^	263,244 tons DM year^−1^	de Vries et al.^55^
			de Jong et al.^54^

### Method of fuzzy cognitive mapping

Fuzzy cognitive maps are fuzzy graph structures for representing causal reasoning^25^. In recent years, FCM has emerged as a valuable method for incorporating stakeholder knowledge into models aimed at understanding the factors contributing to bioeconomy development^27,29,34,51^. In this study, FCM was chosen as a participatory tool to capture and analyze the complex interdependencies between socio-economic, environmental, technological, and political factors influencing the transition to a regional grass-based bioeconomy. FCM enables stakeholders to visually represent their perceptions of how these factors interact, allowing us to identify key drivers, barriers, and feedback loops within the system^56^. The term "fuzzy" refers to the process by which stakeholders assign weights to evaluate the influence and interconnectedness of these factors. The use of FCM not only provided a structured framework but also facilitated stakeholder engagement, ensuring that diverse perspectives were incorporated into the analysis.

**Fig. 2:**
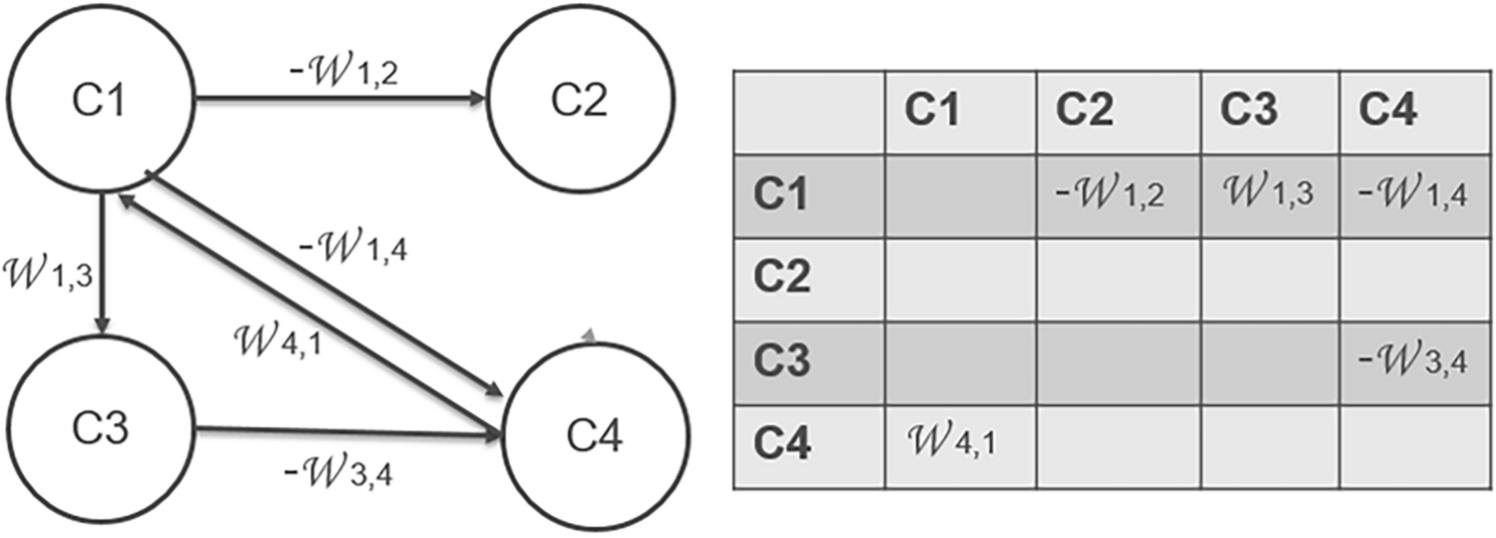
A simple example fuzzy cognitive map comprising four concepts: C1, C2, C3, and C4, along with their associated positive and negative weights.

In the FCM model framework, each concept (C_1_, C_2_, …, C_j_) is visually represented as a node, while the connections between factors are depicted as edges or arrows linking these nodes. These arrows symbolize assumptions about causal relationships and attribute varying values to weigh the strength of each relationship, denoted as W_ij_, with values ranging from − 1 to 1. Positive signs (W_ij_ > 0) indicate that an increase in one node leads to an increase in the linked node, while negative signs (W_ij_ < 0) signify that an increase in one node results in a decrease in the linked node. A weight of W_ij_ = 0 indicates the absence of a causal edge in the graph. It's important to note that these causal weights reflect the opinions of knowledge holders, their explanatory models, and theories of change, rather than constituting predictive statistical models^57^. Figure 2 illustrates a simple example of a FCM with four concepts. Comparing the maps generated by various stakeholders, FCMs can reveal both the similarities and differences between alternative explanatory models and theories of change^58^. Moreover, FCMs can substantially enhance integrated assessment applications by complementing quantitative model approaches with qualitative insights obtained from a structured stakeholder engagement process^59^.

#### Data collection

Between October and November 2022, we conducted semi-structured interviews with the participants, integrating the FCM exercise into the process. Each interview session spanned approximately 1.5–2 h. Directly with the interviewees, each cognitive map was created. Subsequently, we transcribed the interview data gleaned from these sessions and the individual cognitive maps. Additionally, we performed triangulation with both the collected data and the resulting cognitive maps. The selection of participants was carried out using snowball sampling, a non-probability sampling method in which current participants identify additional candidates. While snowball sampling has inherent limitations, such as the risk of bias, it is an effective approach for recruiting individuals with specific expertise and experiences^60^. To mitigate potential biases, including those associated with an unrepresentative initial sample, the sampling process was initiated with a diverse set of stakeholders to minimize path dependencies and enhance representativeness (as indicated in supplementary material Table S3). In the end, we interviewed nine participants, this includes experts from the paper and pulp industry, local farmers involved in grass biomass production, owners of paper manufacturing and printing companies, representatives from local government bodies, consultants specializing in local bioeconomy development, and researchers focused on technology development.

#### Ethical approval

The data for this study was collected through face-to-face interviews, with no experimental procedures involved. All data collection methods adhered to the relevant regulations and guidelines, specifically REGULATION (EU) 2016/679 OF THE EUROPEAN PARLIAMENT AND OF THE COUNCIL, concerning the protection of natural persons regarding the processing of personal data and the free movement of such data. The data collection protocols followed the European Union’s GDPR principals. To ensure participants’ privacy, all personal data was anonymized, and identifying details were coded to maintain confidentiality. Furthermore, informed consent was obtained from all participants for the publication of any identifying information included in the study.

#### Drawing the integrated maps

**Fig. 3:**
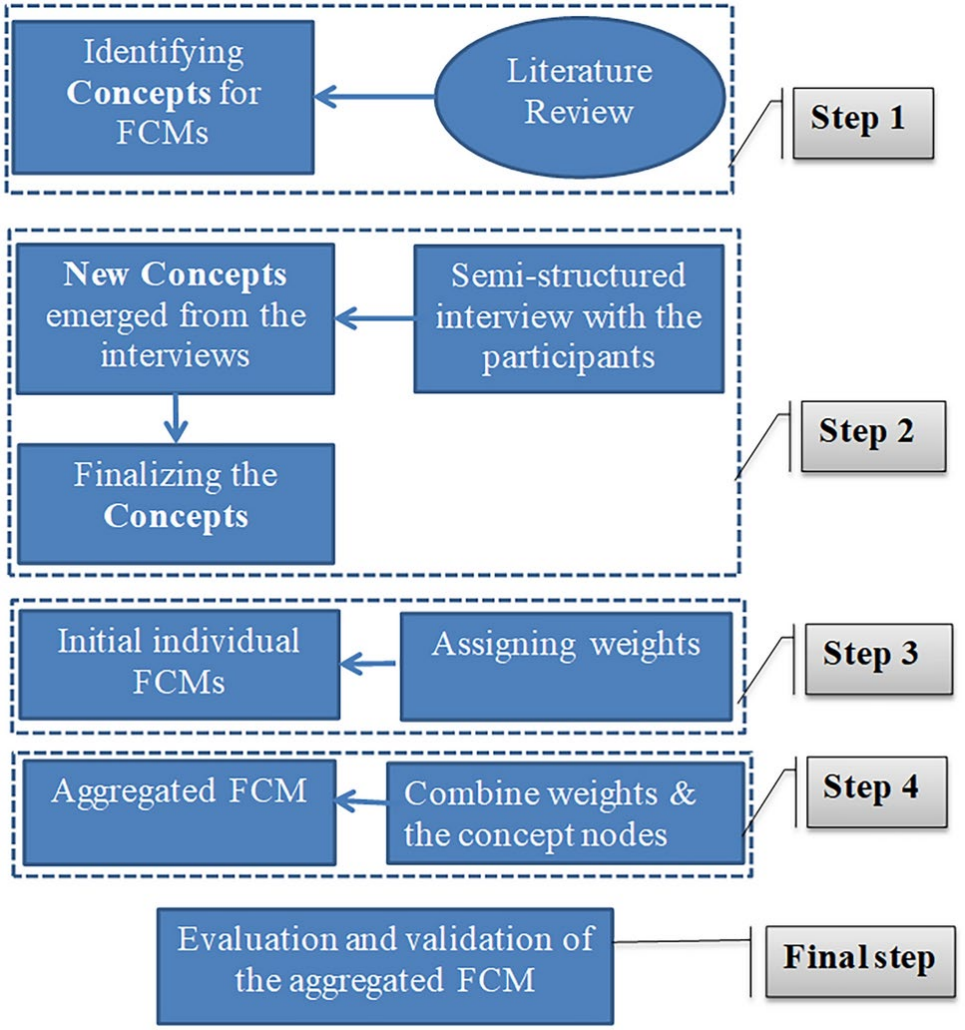
Steps of the FCM development process.

There are three fundamental stages involved in constructing FCM maps, following the methodology outlined by^61^. The process of developing our FCMs is depicted in Fig. 3*.* Firstly, we identified indicators in Step 1 and Step 2. We utilized a literature review to compile a list of factors pertinent to the development of a regional bioeconomy system using residual roadside and natural grass for paper production as indicated in Supplementary material Table S4. Through our semi-structured interviews, new concepts emerged, which we subsequently included in the final aggregated maps. To streamline complexity, concepts sharing similar meanings were clustered together (see the details in the supplementary material).

Next, in Step 3, we constructed individual cognitive maps with various stakeholders. During this process, interviewees assigned weights to factors based on different weight scales provided to them (as detailed in the supplementary material).

In Step 4, the objective is constructing the integrated FCM, taking into account overlapping concepts across individual maps. In this step, an extended FCM map was constructed by aggregating the different FCM maps of individuals to obtain an overarching FCM map that represents the total cognition of all participants. Individual FCMs may share common concepts; they may propose two or more weights for the same interconnection. In such cases, we compute a new weight by averaging the sum of the existing weights as indicated in the literature^62^. This process helps to consolidate the various perspectives represented in the individual maps into a unified model.

Finally, the analysis of the maps was conducted and interpreted using specific tools. We utilized FCMapper (https://www.fcmappers.net/joomla/index.php), a purpose-built software for FCM developed by researchers, which has also been employed in prior publications such as by Morone et al.^29^. Additionally, we used Mental Modeller (https://www.mentalmodeler.com/), a free browser-based software for FCM that has been widely utilized in academic research. These tools facilitated the analysis and interpretation of the FCMs, enabling a comprehensive understanding. The integrated FCM was evaluated based on a range of structural indices, including density, centrality, and their level of complexity. Variables were categorized into three types: ordinary, driver, and receiver. Drivers are variables with forcing functions or "givens," receivers are the "ends" or outcomes, and ordinary variables act as the "means" connecting drivers to receivers. The density of a map reflects its level of connectivity, indicating whether the system is sparse or highly interconnected. Centrality measures the total number of links entering or exiting a variable, along with the weight of these connections, providing insights into the variable's importance within the system^63^. The out-degree and in-degree indices delineate the collective connectivity of nodes, respectively measuring the sum of outgoing and incoming connections. These metrics are derived by aggregating the absolute values of the associated matrix's row and column sums. Centrality, visualized through node size, serves as a gauge of map complexity, elucidating the interconnections between variables and the cumulative intensity of these associations^26^. The level of complexity is derived from the make-up of the different kinds of variables in the system; a high number of receiver variables indicate more considerations about potential results or consequences of system structure and leads to a more complex map. The scenarios are modelled by clamping one or more variables in an iterative process. This provides an indication of how the other components within the system will change under the modelled scenario. To compare the baseline and simulated results, we obtained the steady-state outcome after at least 20 iterations without intervention, starting with initial state vectors set at 1. This steady-state outcome, where there is no change in any of the parameters, serves as the baseline for interpretation. Relative changes in percentage between this baseline scenario and the new steady state are assessed to interpret the results. During the simulation process, a value of 0 indicates the absence of a given concept in the system at a particular iteration, while a value of 1 signifies the maximum presence of the concept^26^. Additionally, a value of 0.1 denotes the minimum presence of the concept. To facilitate sensitivity analysis, values at 0.75, 0.5, and 0.25 are also incorporated for comparison.

## Results

The results of the first part showed the factors affecting the development of paper production from grass fibers. The factors reflect the cognitive awareness of the stakeholders and experts participating in the interviews as well as from the literature. All categories, subcategories, and themes based on content analysis as well as the weights have been illustrated in Table 2*.*

**Table 2:** Descriptive analysis from the constructed FCMs.

Concepts		Out degree	In degree	Centrality	Driver	Ordinary
EC1	Locally sourced feedstock availability	1.08	0.84	1.92		✓
EC2	Competition of feedstock	1.12	1.01	2.13		✓
EC3	Rising wood cellulose price	1.16	0.00	1.16	✓	
EC4	New competitive circular business development	0.44	1.80	2.25		✓
EC5	Economic feasibility	0.95	0.43	1.38		✓
EC6	Production cost	0.54	0.19	0.73		✓
EC7	Energy crisis	0.75	0.00	0.75	✓	
EC8	Niche market formation	0.53	0.37	0.90		✓
SO1	Stakeholders participation	1.12	0.17	1.28		✓
SO2	Farmers' perception of sustainability	0.46	0.83	1.29		✓
SO3	Public awareness	0.98	1.05	2.03		✓
SO4	Public acceptance and willingness to pay	0.61	0.70	1.31		✓
SO5	Fear and risk	0.46	0.69	1.14		✓
SO6	Knowledge gap in sustainable biomass production	0.12	0.33	0.45		✓
PO1	Regulatory impact on waste stream management	0.67	0.00	0.67	✓	
PO2	Supportive governmental sustainable procurement projects	0.55	0.18	0.73		✓
PO3	Financial support and funding	0.68	0.23	0.91		✓
PO4	Promote R&D and cooperation	1.51	0.79	2.31		✓
TL1	Technology optimization and integration	0.95	0.37	1.32		✓
TL2	Technical feasibility challenges for scaling up	0.84	0.09	0.93		✓
EM1	Environment benefits: nature conservations	0.16	0.42	0.57		✓
EM2	Product life cycle management/performance	0.97	0.06	1.02		✓
SE1	Sustainable industry practices	0.34	0.29	0.63		✓
SE2	Prevalence of wood-based paper in the printing sector	0.13	0.04	0.17		✓
SE3	Regional innovation network	1.29	0.13	1.42		✓
SE4	Non-wood fiber-based paper production	0.36	7.75	8.11		✓

EC1–EC7 represent economic factors; SO1–SO6 categorize social factors; PO1–PO4 correspond to policy factors; TL1 and TL2 indicate technological factors; EM1–EM2 denote environmental factors; and SE1–SE4 refer to sectoral factors.

### Descriptive analysis of the constructed fuzzy cognitive maps

As presented in Table 2, our study identifies a comprehensive set of 26 system variables through an extensive literature review and in-depth interviews. These variables cover various aspects of the system and are categorized into six main groups: economic (EC1–EC8), social (SO1–SO6), technological (TL1 and TL2), environmental (EM1 and EM2), sectoral variables (SE1–SE4), and policy and regulation (PO1–PO4). Economic factors, including feedstock availability, competition, rising costs, circular business development, economic feasibility, production costs, energy crises, and niche market formation, collectively influence profitability, sustainability, and competitiveness in resource-dependent industries. Social factors such as stakeholder participation, public awareness, sustainability perceptions, willingness to pay, fear of risks, and knowledge gaps play a key role in shaping the acceptance and adoption of sustainable biomass practices. Optimizing and integrating technologies, along with overcoming challenges related to scaling up their feasibility, are vital for promoting sustainable paper production practices. Environmental benefits, such as nature conservation and effective product life cycle management, play a crucial role in minimizing ecological impacts and ensuring long-term sustainability. Regulations on waste stream management, government support for sustainable procurement, financial incentives, and the promotion of R&D and collaboration are essential policy drivers for advancing sustainability in industries. The aggregated network comprises 107 connections between these system variables. Table 2 provides a comparative analysis of the centrality levels and out- and in-degrees of the different indicators. The aggregated network exhibits a density index of 0.158284, which is lower than that of the original individual maps, which range from 0.20 to 0.40. A lower density index for the aggregated network is due to the increased number of variables included. This indicates that only 15.8% of the maximum potential connections among the 26 concepts are realized.

The findings reveal that SE4 (Non-Wood fiber-based paper production) exhibits the highest centrality, registering at 8.11, indicating its significant interconnectedness from the stakeholders' standpoint. Stakeholders perceive the transition to non-wood fiber-based paper production as potentially beneficial for regional bioeconomy development, particularly through the valorization of residual grass biomass. This notable centrality stems primarily from its comparatively high in-degree index, signifying a considerable influence from other network elements. This observation aligns with the inherent complexity associated with transitioning to a localized bioeconomy system.

Furthermore, several other variables demonstrate relatively elevated centrality values: EC2 (The competition of the feedstock), EC4 (Development of competitive business models), SO3 (Public awareness), and PO4 (Promote R & D and cooperation) recorded centrality scores of 2.13, 2.25, 2.03, and 2.31, respectively. Consequently, these variables play pivotal roles in facilitating the regional bioeconomy transition owing to their heightened sensitivity to changes.

**Fig. 4:**
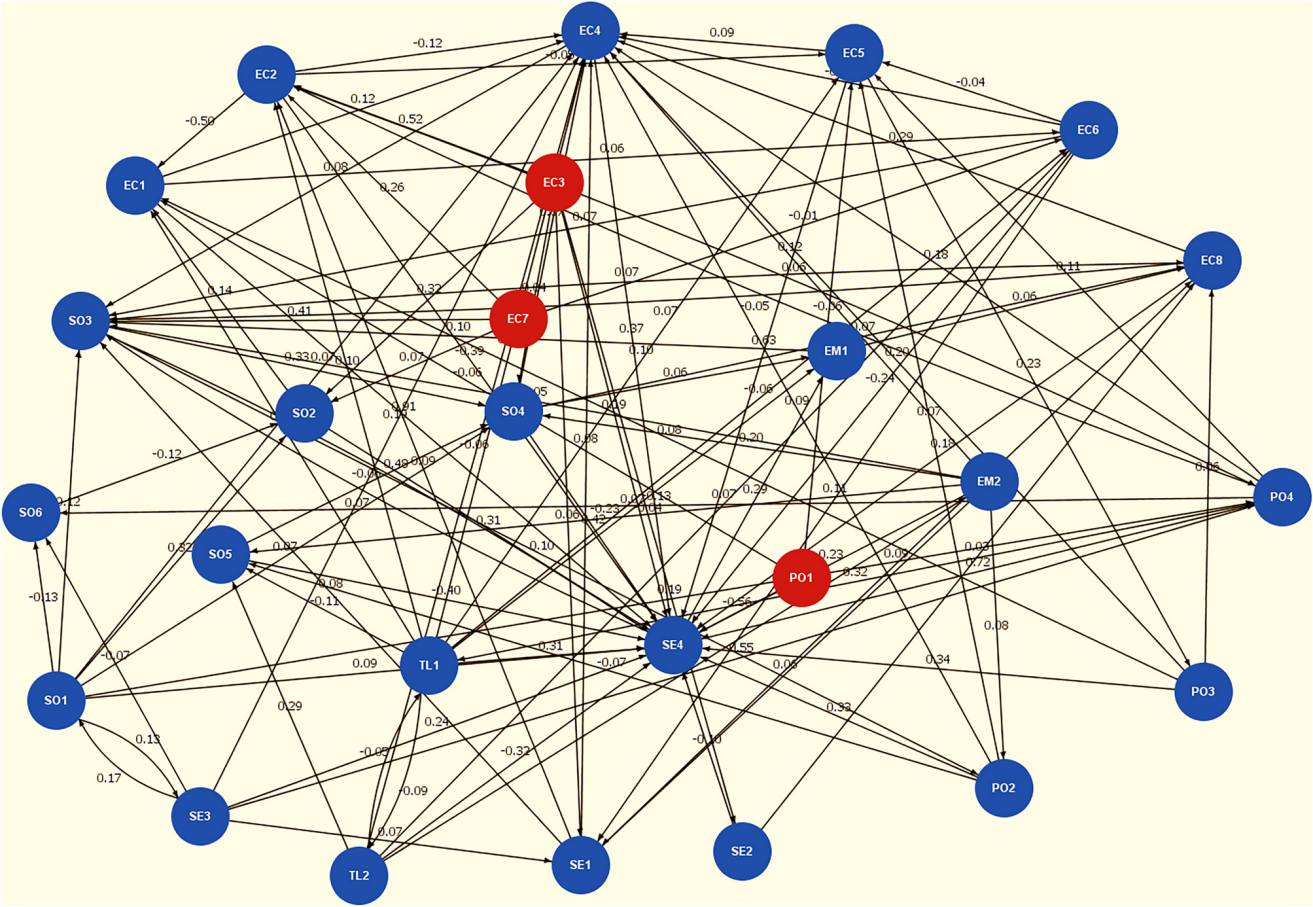
Integrated fuzzy cognitive maps. The figure shows integrated fuzzy cognitive maps with averaged weights from individual maps. Three key drivers are highlighted in red: EC3, EC7, and PO1. The remaining factors, shown in blue, are considered ordinary factors.

Moreover, our analysis recognizes three key system drivers: EC3 (Rising wood cellulose price), EC7 (Energy crisis), and PO1 (Regulatory impact on waste stream management) as shown in Fig. 4. Although these drivers exhibit relatively lower centrality indices, they contribute significantly to the system dynamics. Notably, economic conditions and political variables exert substantial influence on the system, reflecting stakeholders' confidence in their capacity to drive the regional bioeconomy transition by leveraging residual grass biomass for paper production and promoting local economic growth^44^. Conversely, social factors demonstrate heightened reliance on other variables, as evidenced by a greater number of incoming connections compared to outgoing connections.

While centrality-based feature importance methods offer valuable insights into the significance of concepts within a network, they overlook the dynamic nature of the network itself^64^. As such, relying solely on centrality-based approaches may not provide a comprehensive understanding of concept relevance. Therefore, in the following section, we will perform a dynamic analysis of the system variables, examining the interconnectedness of different clustered categories with other variables, and conduct a what-if analysis to create both best and worst-case scenarios to observe potential system changes.

### Scenario analysis of the constructed regional grass-based bioeconomy system

**Fig. 5:**
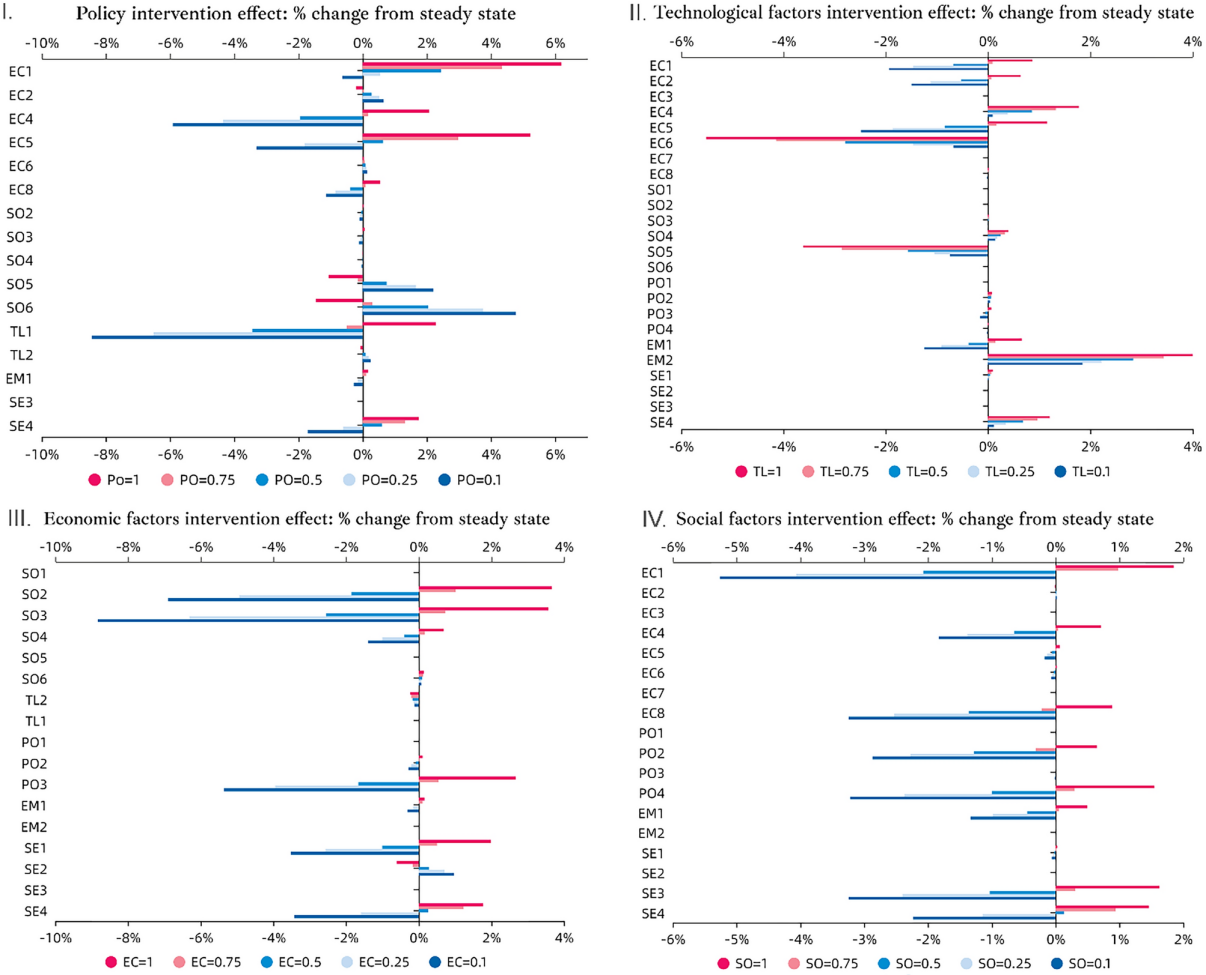
Illustrates simulation results depicting the best and worst-case scenarios. (**I**) Demonstrates the system's change resulting from policy factors (PO1, PO2, PO3, PO4). (**II**) Indicates the system's change from technological factors (TL1, TL2). (**III**) Displays the intervention effect of economic factors (EC1–EC8) on the entire system. (**IV**) Illustrates the intervention effect of social factors (SO1–SO6) on the system.

Figure 5 illustrates the results of the what-if analysis. Figure 5I pertains to the simulation of all policy and regulation concepts to respective values of 1, 0.75, 0.5, 0.25, and 0.1. Similar simulations are made for economic, social, and technological factors, enabling comparative analysis across different scenarios. The rationale underlying the classification of these system variables into categories such as policy and regulation, economic, social, and technological is explicated in Fig. 5. This figure elucidates the potential impact of these factors on the overall system structure.

In general, as depicted in Fig. 5I, the political and regulatory dimensions, including PO1 (Regulatory impact on waste stream management), PO2 (Supportive governmental sustainable procurement projects), PO3 (Financial support and funding), and PO4 (Promotion of R&D and cooperation), exhibit a dynamic influence on the entire system, particularly on economic, social, and sectoral factors. The strong presence of these political dimensions correlates positively with economic factors such as "Locally sourced feedstock availability", where the adoption of a mixed policy approach is projected to result in a more than 6% increase from the baseline scenario. Conversely, a low presence of supportive policies (below 0.75) negatively influences "Competitive circular business model development," potentially hindering sector growth and investment, as well as the "Economic feasibility" of initiatives. Regarding social factors, the robust implementation of these political dimensions (PO = 1) is expected to address societal concerns, such as reducing "Fear and risk" and bridging "Knowledge gaps on sustainable biomass production" leading to a 2% improvement compared to the baseline scenario. The significant presence of these political concepts correlates with positive impacts observed on economic factors, such as "Locally sourced feedstock availability", "Competitive circular business model development" and "Economic feasibility". Regarding social factors, the presence of these political dimensions is anticipated to mitigate societal concerns related to "Fear and risk" and "Knowledge gaps on sustainable biomass production".

Additionally, technology factors, such as "Technology optimization and integration" stand to benefit from this presence, albeit to a lesser extent. Conversely, a lower level of presence of these concepts may negatively affect technology factors, as current technological development may require substantial governmental support, particularly financially. Furthermore, the PPI is also expected to benefit from strong presence of these political and regulatory initiatives, with positive percentage changes observed, nearing 2%. Upon a closer examination of the changes resulting from reducing the low-level presence of these policy initiatives (PO = 0.1), stronger negative impacts (− 2%) become apparent. This underscores the importance of maintaining a strong political presence to facilitate the transition effectively.

When examining the technological dimension of the system as indicated in Fig. 5II, the significant presence of "Technology optimization and integration" and "Technical feasibility challenges for scaling up" yields substantial influences, primarily clustered around economic factors. "New competitive circular business development" shows positive progress, with the highest increase of 2% under a strong presence of technology development (TL = 1). Similarly, "Production cost" benefits significantly from technology optimization, with production costs reduced by 5% when technology levels are fully optimized (TL = 1). These advancements are perceived positively by stakeholders, as they enhance economic viability and reduce barriers to scaling up sustainable technologies. Moreover, technological advancements reduce societal fear and risk associated with utilizing roadside grass as a product source, further reinforcing stakeholder confidence in sustainable practices. Additionally, technological factors influence "Product life cycle management/performance" contributing positively to carbon emission reduction and overall environmental benefit. Despite the positive contributions of technological advancements, their influence on sectoral factors, particularly in utilizing roadside and nature grass-based biomass for paper production, is limited compared to political dimensions. The highest observed change from the steady-state outcome to the simulated results, under the maximum presence of technological factors, is at 1.2%.

All illustrated in Fig. 5III, the economic clustering factors exert a significant influence on social, political, and sectoral dimensions, contingent upon the robust presence of economic conditions. Conversely, a diminished presence of these conditions (when SO ≤ 0.5) may lead to adverse effects, particularly evident in indicators such as "Farmers' perception of sustainability", "Public awareness" and "Public acceptance & willingness to pay". For instance, a negative impact of up to 9% compared to the steady-state value is observed for the factor "Public awareness" in the absence of strong economic conditions, such as economic feasibility, feedstock availability, niche market formation, and the development of competitive circular business models. These economic factors are crucial for driving the transition toward a local circular bioeconomy utilizing grass biomass for paper production. When these economic factors are highly presented, they positively influence public perception and awareness, increasing it by 4%. Additionally, a significant decrease of 3.5% from the steady-state value in the sectoral transition to grass paper production is observed under minimal economic conditions (EC = 0.1). Stakeholders consistently prioritize economic factors as the most critical drivers of this transition, often outweighing the perceived impacts of political and technological factors.

When scrutinizing the social dimension of the system as shown in Fig. 5IV, its effect extends to economic, political, and sectoral factors, particularly affecting indicators such as "Locally sourced feedstock availability", "Competitive circular business model development" and "Niche market formation". Additionally, at the policy level, the presence of factors such as "Supportive governmental sustainable procurement projects”, and “Promote R&D and cooperation” as well as at the sectoral level, "Regional innovation network" and "Non-wood fiber-based paper production", is influenced. The absence of critical social factors, such as stakeholder participation, public awareness, and knowledge on sustainable biomass production, negatively affects the system's dynamics. For instance, locally sourced feedstock decreases by 5.3% from the baseline scenario, while niche market formation experiences a decline of 3%. Additionally, a low presence of these social factors (SO < 0.75) significantly undermines regional innovation networks, leading to a 3.5% decrease compared to the baseline. Conversely, a strong presence of social factors promotes sectoral transitions, driving a 1.5% increase in grass paper production. Although this impact is slightly less pronounced than that of economic factors, the integration of social factors remains essential in facilitating positive transitions. A lack of robust social support risks stalling the transition process, reinforcing the complexity of social, economic, political, and sectoral dimensions. In summary, these observations reaffirm our research question by highlighting the interconnectedness of social, economic, political, and sectoral dimensions in the transition toward a local circular bioeconomy centered on grass-based innovations.

**Fig. 6:**
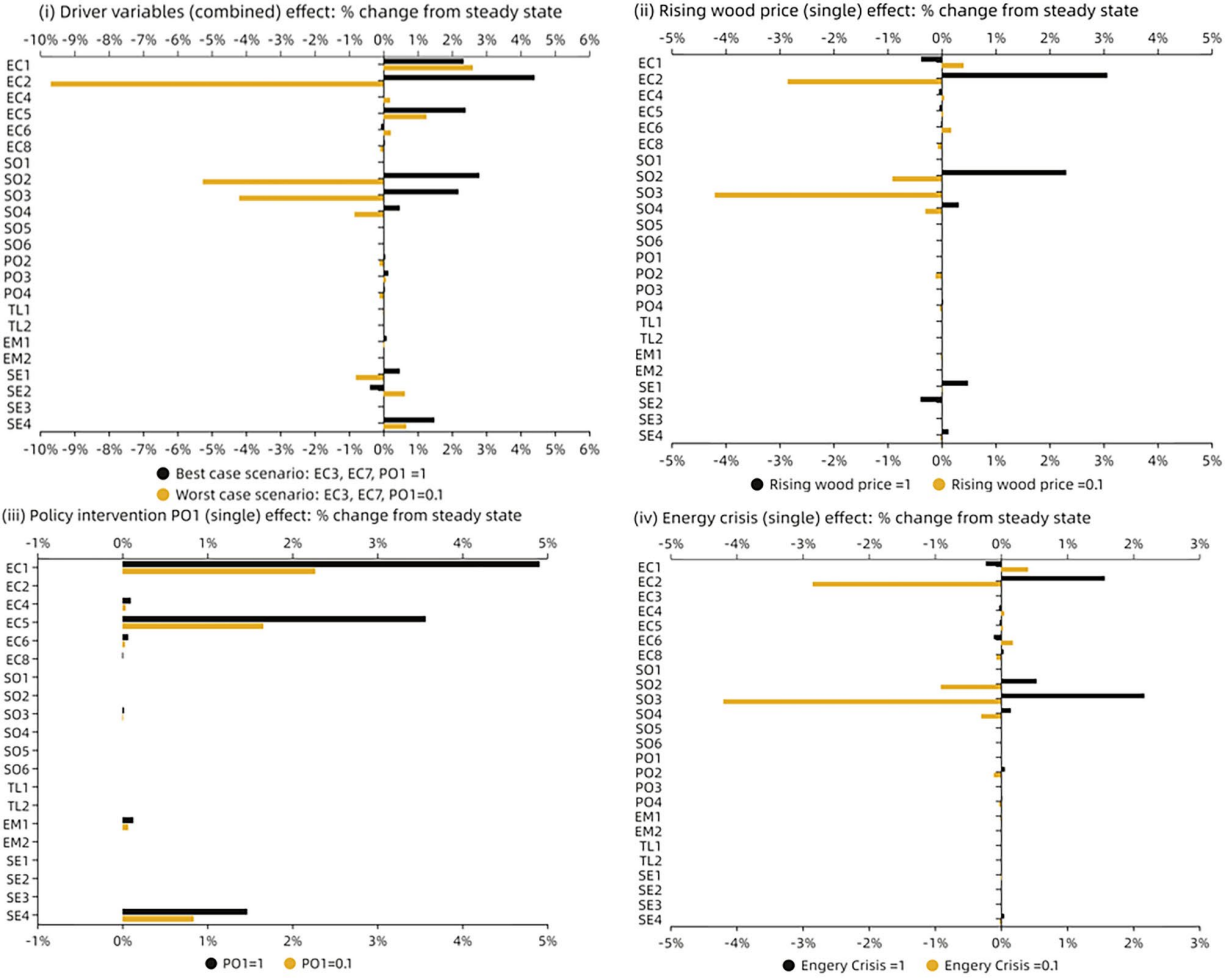
Depicts the best and worst-case scenarios regarding the identified system drivers (EC3, EC7, and PO1) (**i**) Shows the combined effect of the three drivers on the system; (**ii**) Illustrates the impact of rising wood prices on the entire system; (**iii**) Examines the influence of policy drivers on the system; (**iv**) Analyzes the impact of the energy crisis as a driver on the system.

Continuing the analysis, for answering our first research question on the system drivers, we delve into the effects of identified drivers by stakeholders on the entire system, considering both their combined and individual impacts. The identified drivers encompass "Rising wood price”, "Energy crisis" and "Regulatory impact on waste stream management". In general, as depicted in Fig. 6i, these drivers predominantly influence economic, social, and sectoral categories, particularly in scenarios representing both best and worst cases. Indicators such as "Locally sourced feedstock availability", "Competition of feedstock", "Farmers' perception on sustainability", "Public awareness" and "Non-wood fiber-based paper production" exhibit notable effects. Notably, the "Competition of feedstock" indicator displays significant sensitivity to the presence of system drivers. In instances of strong drivers’ presence, intensified competition for feedstock arises, whereas low driver presence diminishes such competition. This phenomenon aligns with the rationale that rising wood prices, especially amid an energy crisis, prompt industries to seek alternative biomass sources, thereby intensifying competition for feedstock used in paper production.

Examining the individual effects of each driver, demonstrated in Fig. 6ii, iii, and iv, reveals distinct impacts on social factors. Interestingly, the rising wood price directly influences farmers' perceptions of biomass sustainability. Conversely, the energy crisis amplifies public awareness of sustainable development issues. Higher energy prices resulting from the crisis prompt consumers to reconsider sustainability, thereby positively influencing public awareness.

## Discussion

In the result section, we have identified the influential role of political support and regulatory initiatives in driving the transition towards on-wood fibers for paper production. This aligns with previous research by Ladu et al.^27^ and Morone et al.^29^, suggesting that a policy mix incorporating economic and financial support for sustainable bioeconomy is likely to yield the most favorable outcomes. Dedicated policies could provide a balanced push for the bio-based economy transition towards a circular and innovative trajectory. When policies aimed at promoting sustainability are weakened or absent, the potential for negative consequences on economic, social, and technological factors becomes evident. Therefore, sustaining political engagement and regulatory support is imperative to realize the desired transition towards a sustainable bioeconomy. In addition, understanding the interplay of coordination, timing, and scale in policy mixes is essential for grasping how different instruments can accelerate sustainability transitions. The Swedish paper and pulp industry exemplifies the need for destabilizing policies, such as stringent environmental regulations, to precede innovation policies. These initial measures create an environment where novelty creation policies can effectively drive industry transformation^65^.

Furthermore, the case study under examination revolves around an immature technology still in its nascent phase. Early-stage technology development can be affected by economic activity. During economic downturns, there is often less funding available for policies supporting new technology development, as public funds are limited. Stakeholders may worry that funding will be prioritized for activities with immediate economic impact rather than for long-term technological advancement. This highlights the importance of policymakers ensuring consistent support for early-stage technology development, even during economic uncertainty, to foster innovation and economic growth over time^66^. Transitioning to sustainability also requires a balanced politico-economic framework. Gawel et al.^67^ argue that achieving sustainability relies on finding the right balance between regulatory frameworks and market mechanisms to implement necessary transition policies. Effective policy interventions and market regulations play a pivotal role in steering the transition towards sustainability and ensuring that technological advancements align with overarching societal objectives. This assertion is supported by research findings highlighting the significance of political factors in driving the development of technology aimed at valorizing biomass. Specifically, these political influences are identified as primary drivers for overcoming barriers, demonstrating a greater impact compared to socio-technical factors^36^. This correlation aligns with our findings. Therefore, it underscores the critical importance of implementing robust policy frameworks and regulatory mechanisms to effectively align technological advancements with sustainability goals^68^.

Aligned with Rajeswar^69^, our findings suggest an inclination towards overestimation of the sustainability gains associated with novel technologies, juxtaposed with an under-exploration of complex and potentially adverse impact pathways during early innovation phases. It is often argued that excessively regulated innovation systems might impede much-needed technological advancement. Nevertheless, this should not serve as a justification for neglecting thorough scrutiny in technology impact assessments^70^. The development of more sophisticated models to assess and integrate industry-based technology is required^71^. Our results indicate that while economic incentives are strongly connected to other development factors, their application alone may lead to unintended consequences, such as environmental degradation from resource overexploitation. This finding highlights the limitations of relying exclusively on economic incentives to drive sustainability transitions within this system. The transition to grass-based paper production represents a promising eco-friendly alternative to traditional paper^72^. However, its adoption and commercial viability are hindered by higher production costs and functional differences compared to conventional paper. These cost disparities, coupled with broader industry dynamics, restrict grass-based paper to a niche market rather than positioning it as a feasible mainstream alternative^44^. This underscores the need for a more comprehensive approach that addresses economic, environmental, and functional challenges to promote sustainable innovation effectively.

From our simulated results, it is evident that stakeholders perceive a significant competition for utilizing grass biomass for various purposes, particularly in light of concerns surrounding the European Union's energy security following the Russian-Ukrainian conflict. The conflict has highlighted the EU's heavy reliance on imports of raw materials from these countries, emphasizing the need to develop alternative sources or renewable raw materials for material use and energy production and to reduce imports of raw materials from conflict regions^73^. The potential utilization of roadside grass for bioenergy production, as highlighted by Meyer et al.^15^ and Ravi et al.^74^, presents a promising opportunity. In the face of this competition for biomass use across different sectors, policymakers should focus on establishing clear guidelines for biomass utilization. This is especially important given the institutionalization of bioeconomy strategies at the European level. These strategies provide a framework for developing cohesive approaches to biomass utilization that consider both environmental and economic factors.

Additionally, the energy crisis between 2021 and 2023 has catalyzed increased social awareness among the public, underscoring the significance of nurturing and fostering robust social factors to drive this transition effectively. This emphasizes the need for comprehensive strategies that take into account the interplay between social, economic, and political dimensions. For a successful transition, it is essential to adopt both top-down and bottom-up approaches^44^. This involves engaging stakeholders at various levels of governance and society, ensuring inclusivity, and promoting collective action towards sustainable solutions.

### Limitations and future studies

While this study provides valuable insights, it has several limitations that should be acknowledged. First, the use of FCM is challenged by the relatively small number of participants involved, which may limit the representativeness of the findings^75^. Additionally, the study focuses on a single country case, making it difficult to illustrate the broader global importance and applicability of the system under investigation. Expanding the scope by incorporating additional factors and insights from other expert knowledge could enrich the analysis. Future research could implement this method across diverse country settings, enabling a comparative analysis of the results obtained. Although the relationships between factors were explored, they may not fully capture the complexity of the system, and periodic updates with new data could enhance the accuracy of the model. However, acquiring and processing new knowledge is inherently time-intensive, posing a practical challenge^75^. Future research on FCM should address issues of reliability and validity more rigorously, providing transparent approaches for quality assurance. These improvements would contribute to strengthening the robustness and applicability of FCM in similar contexts. Second, exploring additional scenarios beyond those examined in this study could provide deeper insights into the dynamics of the system. While this study emphasizes the importance of policy mixes in accelerating sustainability transitions, it also has several limitations. The implementation of proposed policy frameworks is complex and can vary significantly across different regulatory and economic contexts. Moreover, the study does not address the associated costs of these policy changes, including both short-term financial burdens and long-term economic impacts. Future research should prioritize quantifying these costs to offer a more comprehensive understanding for strategic planning. This financial perspective is critical for informing decisions by policymakers and industry stakeholders. Third, technological advancements, such as improved biomass processing techniques and optimized resource allocation, hold significant potential to enhance economic viability. Future studies could focus on integrating these innovations to bolster cost-efficiency and scalability. Lastly, the study only partially addresses the necessity of continuously adapting and revising policies in response to the evolving nature of sustainability transitions. Future studies should adopt a more iterative approach to policy development, integrating ongoing changes and feedback mechanisms within the industry to better navigate the dynamic landscape of sustainability transitions.

## Conclusion

This study significantly contributes to the discourse on decarbonizing the paper and pulp industry by applying fuzzy cognitive maps to capture and model local stakeholders' perceptions of transitioning to a local bioeconomy using roadside and natural grasses for paper production. The analysis reveals the pivotal role of political support and regulatory initiatives, with key drivers including rising wood prices, energy crises, and regulatory impacts on waste stream management. Political interventions are particularly influential at the regional level, emphasizing the need for comprehensive policy frameworks that address socio-economic, political, environmental, and sectoral barriers. Technological advancements, though beneficial for economic viability and reducing societal fears, have a lesser impact compared to political dimensions. Economic factors are prioritized by stakeholders and significantly influence public awareness and acceptance. The interconnectedness of social dimensions with other factors further highlights the complexity of the transition. Recommendations for policymakers include enhancing stakeholder engagement through inclusive top-down and bottom-up approaches, ensuring clear and adaptive guidelines for biomass utilization, and sustaining financial support for early-stage technologies. It is essential to prioritize ongoing assessment of technology impacts to inform evidence-based policy decisions effectively. Future research should focus on quantifying the broader implications of policy changes to provide a clearer strategic outlook and emphasize the novel insights gained in the context of advancing sustainability within the paper and pulp industry.

## Supplementary Information

Supplementary Information.

## Data Availability

The data that support the findings of this study are available from the corresponding author upon reasonable request.
