# Supplementary material for: Understanding system interdependencies in sustainable paper production from residue grass biomass: Insights from fuzzy cognitive mapping
Source: Sci Rep. 2025 Jan 9;15:1398. doi: 10.1038/s41598-024-84358-4 (PMC11717917; doi:10.1038/s41598-024-84358-4)
Supplement: Supplementary file 1 — Supplementary Information. [file 41598_2024_84358_MOESM1_ESM.pdf]

This is a supplementary file for the article published in Scientific Report titled:

Understanding System Interdependencies in Sustainable Paper Production from Residue Grass Biomass: Insights from Fuzzy Cognitive Mapping

Zhengqiu Ding[a, b,*], Philipp Grundmann[a, b]

[a] Innovations in Sociotechnical Systems, Department of Technology Assessment, Leibniz Institute for Agricultural Engineering and Bioeconomy (ATB), 14469 Potsdam, Germany

[b] Department of Agricultural Economics, Humboldt-Universität zu Berlin, 10117 Berlin, Germany

[*] Correspondent author: zding@atb-potsdam.de

Supplementary Material Introduction: Interview Guidelines and Participants

This supplementary material includes the interview guidelines and detailed steps for conducting fuzzy cognitive mapping (FCM) with stakeholders regarding the use of roadside and natural grasses for paper production. The guidelines outline the structured approach taken during the interviews to gather comprehensive and relevant data, ensuring the effective capture of stakeholder perceptions and insights. The process described here is critical for understanding the multifaceted dynamics and influences within the local bioeconomy transition.

1. Interview guideline

This interview aims at collecting information related to the internal and external factors supporting or limiting the development and implementation of novel technologies tested at demo sites valorising the roadside grass and nature grass for a regional bioeconomy.

Basic information

- Geographical location (Country and area)
- Occupation/title of interviewee?
- Approximate size of business (if possible: annual turnover and area of farmland)
- What are the main products related to your business?
- What type of company to you work for (large corporation, family owned etc)?
- Have you even worked with grass-based businesses? (Yes/no)

Part 1: Questions related to your view on the grass-based innovation

These questions are meant to form an understanding of the business environment and its role in enabling the development of sustainable business models based on the novel technologies developed for processing the grass fiber for papermaking. The business environment includes the actors (e.g. institutions, knowledge and education providers, funding, infrastructure, consumers etc.) and what activities these stakeholders are involved in in relation to the innovation. The aim is to allow for a mapping of some of the most important aspects that incorporated with development of sustainable business models applies to the technologies implemented at the demos (which will be done in Part 2).

1. In your opinion, what are the most important aspects that incentivises developing and implementing the technology and to promote novel grass-based value chains in your region in general (e.g. biomass availability, improved land use, promising business opportunity)? Why?
2. What are the relevant rules, regulations or policies (e.g. local/national laws, financial support, other support measures related to e.g. biodiversity) that could facilitate or inhibit the development of the technology? How?
3. How would you describe the availability and access to funding (e.g. private funding from entrepreneurs, state funding, EU-based support for farmers) for your potential business?
4. How would you describe the availability and access to resources (grass) and infrastructure availability needed for the technology to function?
5. How would you characterize the consumers/community perception or value towards the new products and service from this technology and innovation? Good/bad/neutral? Why? Is there any way the innovation could be made more attractive?
6. If possible to do so, how would you describe the know-how (e.g. specific ways of farming/handling the grass resource that work/does not work, experience in farming) required to implement the technology in the demos?
7. Will you continue utilising the innovation/business after the end of the demo project? If yes, why, if no, why?
8. Compared to a business-as-usual scenario (i.e. your existing business), what unique barriers exists for implementation of the innovation (e.g. lack of economic support or resources to work on implementation, lack of policies/political support, interest in the innovation)?
9. How would you assess the relationship between the technology developed for making grass paper and the environmental benefits in the following table S1.

Table S1: Impact of technological development on the environment

**Impact of technological development interference on the environment**					
		Positive	Negative	Not relevant	Comment
Provisioning	Forage, milk,meat, fiber				
	Biomass for bioenergy				
	Biomass for biorefinery				
Supporting	Buffering/accelerating/slowing down of nutrient cycling				
	Water infiltration and retention in soil				
Regulating	Erosion control				
	Flooding control				

	Carbon sequestration				
	Greenhouse gas mitigation				
	Wildfire control				
	Water purification				
	Preseving biodiversity				
Cultural	Grassland as herited cultural landcape				
	Leisure activities/recreation				
Other/additional factors					

Part 2: Fuzzy cognitive mapping

This exercise is to recap the information discussed in the Part 1 with the stakeholders (interviewees) and to map the linkages between the factors.

Step 1: Interviewer may help the interviewee recall the elements or factors, which he or she mentioned during the interview in relation to technology implementation for promoting novel grass-based value chains. (Below is an example of the list of factors, which the stakeholder might have mentioned during the interviews, please write down the key word)

Example:

Feedstock availability (grass biomass)
Land availability
Supportive legislations/regulations
Rural population
Finance and Funding
Infrastructures
Knowledge
Bio-based products production
Community acceptance
Technology implementation
Employment/Jobs opportunities
Ecological sustainability
Fossil fuel price
Well-being of the local residents
etc….

Step 2: With all these elements, create a map/network:

a. Place the technology implementation in the centre
b. Connect this to others factors and identify possible connections in-between them

c. Identify the sense of each connection: positive (+) or negative (-)
d. Ask the interviewees set weights for each connection mapped between -1 and +1
(Weights refer to the importance of the cause-effect relation between those two elements. That is, in a scale from -1 to +1, how much influence does the first element have over the second?) You can assign positive weights such as: 0.05, 0.10, 0.15, 0.20 …. and negative weights such as -0.05, -0.1, -0.15, -0.20 ….

Table S2: Guidelines for determining the weights of relationships between factors

**Scale:**	**-1**	**-0.9**	**-0.8**	**-0.7**	**..**	**..**	**..**	**0**	**..**	**..**	**..**	**0.7**	**0.8**	**0.9**	**1**
**Perceived impact**	Very negative impact							No effect							Very positive impact
Lack of grass biomass															
Finance and Funding															
Technology implementat-ion															
Other															

The aim is to assess weights for each relation independently to the others, in a way that they do not need to sum up to 1, but their weights need to be comparable. For this, identifying one reference connection, and setting the weights of the others comparatively to that one, can be a good choice if deemed possible.

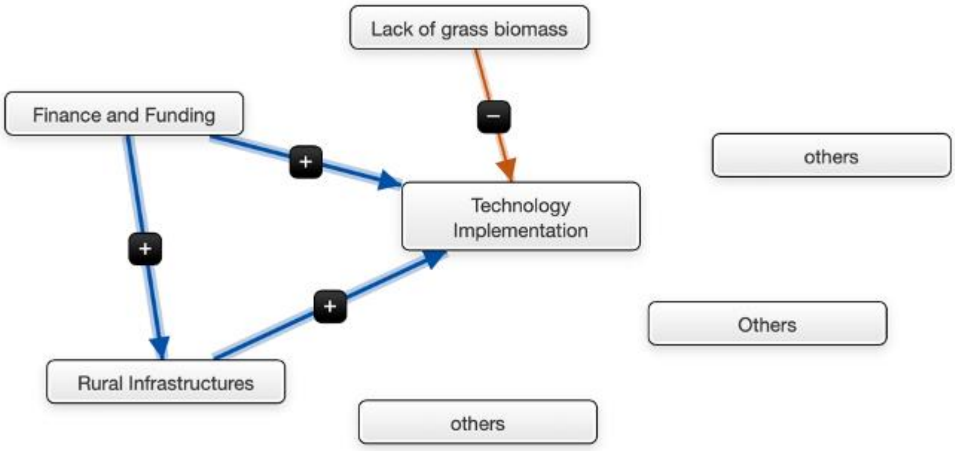

Figure S1: An example of the cognitive map for guide the participant for construct the whole map

(The cognitive mapping exercise will be done in the software **Mental Modeler** and lead by a representative in the workshop (example in image above)).

2. List of the participants

The following table outlines the key stakeholder groups engaged in our study to develop Fuzzy Cognitive Maps (FCM) for transitioning to a local bioeconomy using roadside and natural grasses for paper production. Each stakeholder group was selected to provide diverse perspectives and expertise, ensuring a comprehensive understanding of the socio-economic, environmental, and technological factors involved. Interviews were conducted with representatives from various sectors, including industry, government, research, and non-governmental organizations, to capture their insights and model the complex dynamics of this transition. The table details the stakeholder groups, their roles, the duration of their engagement, and additional notes highlighting their unique contributions to the study.

Table S3: List of participants interviewed and involved in constructing the fuzzy cognitive maps

Stakeholder group	Role	Time duration	Additional notes
Nature conservation association	Business development manager	120 minutes	Focus on sustainability and environmental impacts.
SME (paper making industry)	Manager	100 minutes	Provides insights into operational challenges and innovations.
Paper industry association	Director	80 minutes	Represents industry-wide interests and regulatory compliance.
Bioeconomy consulting	Bioeconomy business consultant	120 minutes	Offers expertise on market trends.
NGO	Manager	100 minutes	Nature conservation and biomass supply
Regional governmental agency	Regional governmental officers for economic development	120 minutes	Focus on regional policy implementation and support.
Research institute	Researchers on technology development	120 minutes	Provides technical insights
Agriculture sector/grassland	Organic dairy farmer	100 minutes	Offers perspective on agricultural practices and biomass supply.
SME (small paper making and printing company)	Owner	100 minutes	Insights into small-scale production and market integration.

3. List of the system variables identified

After conducting interviews and reviewing the literature, we identified 26 system variables. Some overlapping variables were grouped together as the same variable in our process. Table S4 below presents the identified variables along with explanations for each.

Table S4 List of the system variables identified from the literature and with the stakeholders

Concepts	Code	Description	Sources of the Concepts (literature and interviews)
Locally sourced feedstock availability	**EC1**	*Quality and quantity of the locally sourced grass biomass*	(El-Sayed et al. 2020); (Furszyfer Del Rio et al. 2022); (Bajpai 2021)
Competition of feedstock	**EC2**	*Competition on the alternative use of the grass biomass in other sectors, e.g., fermentation process, or as building materials for the construction industry*	Stakeholders: researchers on technology development; manager of the paper-making factory; bioeconomy consultant; Regional officers for economic development; organic dairy farmer; Nature conservation association
Rising wood cellulose price	**EC3**	*With the increase in the cost of wood cellulose since 2020, it is a crucial component in various industries, including paper, textiles, and biofuel production.*	(Bian et al. 2019)
New competitive circular business development	**EC4**	*Establish a competitive regional circular bioeconomy business model with a focus on customer's needs and requirements to ensure the success of the business cases.*	(Furszyfer Del Rio et al. 2022)
Economic feasibility	**EC5**	*The assessment of the investment is financially viable and capable of generating positive economic returns.*	(Bajpai 2021)
Production cost	**EC6**	*High laboring costs associated with the harvesting process of the grass biomass, and pre-processing them, costly transportation of water-containing grass biomass, and energy consumption in processing.*	(Ramdhonee and Jeetah 2017)

Energy crisis	**EC7**	*Rising energy prices in Europe, combined with increasing gas prices, place the sector at a global competitive disadvantage.*	Stakeholder: local government officer on economic development, manager of the paper-making factory
Niche market formation	**EC8**	*Developing a circular business strategy specifically focused on targeting and serving a niche market, e.g., high-quality art paper from grass.*	Stakeholder: consultant on bioeconomy development; Nature conservation association
Stakeholders participation	**SO1**	*Involving stakeholders in decision-making processes to increase transparency, improve acceptance and support, and emphasize the value of diverse perspectives and the potential for building trust and long-term relationships.*	(Ramdhonee and Jeetah 2017)
Farmers' perception of sustainability	**SO2**	*Attitudes, beliefs, and perceptions of local farmers towards adopting new practices related to sustainable grass biomass use.*	Stakeholder: nature conservation association; Organic dairy farmer
Public awareness	**SO3**	*Consumers making conscious decisions to reduce resource consumption minimize waste generation, and prioritize environmentally friendly products e.g., grass-based paper.*	Stakeholders: NGO; Bioeconomy consultant; researchers on technology development; organic dairy farmers; Nature conservation association
Public acceptance & willingness to pay	**SO4**	*Consumers' willingness to pay the premium price for bio-based products.*	(Furszyfer Del Rio et al. 2022)
Fear and risk	**SO5**	*Perceived uncertainties/potential risks using roadside grass to make paper by the public.*	(Furszyfer Del Rio et al. 2022)
Knowledge gap in sustainable biomass production	**SO6**	*The lack of comprehensive knowledge and information related to sustainable biomass production practices is linked to the business cycle by the local farmers.*	(Furszyfer Del Rio et al. 2022)

Technology optimization and integration	**TL1**	*Optimization of the harvesting machinery/technology, Low-Cost Pretreatment Technology (Conserving Grass Biomass), and integration of technology for byproducts production during the process*	(Pari et al. 2015), (Furszyfer Del Rio et al. 2022)
Technical feasibility challenges for scaling up	**TL2**	*Challenges for Technical feasibility and experimentation Challenges, from the experimentation phase to large-scale production*	(Liu et al. 2018)
Regulatory impact on waste stream management	**PO1**	*Current roadside grass by regulation is considered a waste stream, therefore to reuse of this type of biomass should be regulated under the waste streams thus there is a need to change this regulation and allow for extended use. Approval procedures/regulations for individual permits for the use of certain fibers for paper making (if the fiber is produced somewhere else)*	Stakeholders: Paper industry association; researcher on the technology development; manager of the paper-making factory; organic dairy farmer
Supportive governmental sustainable procurement projects	**PO2**	*Policies to integrate environmental criteria into public purchasing processes. Such as Green Public Procurement (GPP) encourages the procurement of products and services with reduced environmental impact throughout their life cycle.*	Stakeholders: bioeconomy consultant; Regional governmental officers for economic development; SMEs; Nature conservation association
Financial support and funding	**PO3**	*The provision of financial resources to enable the implementation of various projects, initiatives, or activities for the adoption of practices that promote environmental, social, and economic sustainability throughout value chains.*	(Furszyfer Del Rio et al. 2022)
Promote R & D and cooperation	**PO4**	*Policies for increasing R&D cooperation between organizations and public research institutions (PPP), Cross-sectors collaboration, and developing partnerships between other organizations from different sectors, such as the public, private, and nonprofit sectors.*	(Furszyfer Del Rio et al. 2022)

Environment benefits: nature conservations	**EM1**	*Reduce CO2 emission, low Carbon Footprint Logistics, and environmental benefits of the products when substitution of wood with sustainably sourced grass biomass*	(Kissinger et al. 2007; Furszyfer Del Rio et al. 2022)
Product life cycle management/ performance	**EM2**	*Research on climate and resource protection successively improves the life cycle performance of the products due to new insights, e.g., after-use disposal, and promotes the circularity of the production system.*	(Sun et al. 2018); (Lai et al. 2023);(Ghose and Chinga-Carrasco 2013)
Sustainable Industry Practices	**SE1**	*Transparent Manufacturing Processes sustainably sourced the biomass for paper production, Transparent Manufacturing Processes*	(Furszyfer Del Rio et al. 2022); (Lai et al. 2023)
Prevalence of wood-based Paper in the printing sector	**SE2**	*Wood fiber-based paper continues to dominate the printing sector due to its abundant availability, cost-effectiveness, and versatility. With its optimized printability, ink absorption, and image reproduction capabilities, it remains the preferred choice for a wide range of printing applications.*	Stakeholder: business owner of the small paper-making factory and the printing company
Regional innovation network	**SE3**	*Recognizes the importance of collaboration, knowledge sharing, and regional synergies to foster innovation, economic growth, and sustainable development within a specific geographic area, for instance, regional bioclusters.*	Stakeholders: bioeconomy consultant; regional government officers on economic development
Non-wood fiber-based paper production	**SE4**	*Non-wood fiber-based paper production involves the creation of paper using alternative fibers that are sourced from materials other than wood. Various sustainable alternatives exist, such as grass fibers, which can be utilized to produce environmentally friendly paper.*	Stakeholder: business owner of the small paper-making factory and the printing company; manager of the paper-making factory, technology developer, organic dairy farmer; NGO; Nature conservation association; Bioeconomy business consultant

References:

Bajpai P (2021) Nonwood Plant Fibers for Pulp and Paper. Elsevier

Bian H, Gao Y, Luo J, et al (2019) Lignocellulosic nanofibrils produced using wheat straw and their pulping solid residue: From agricultural waste to cellulose nanomaterials. Waste Management 91:1–8. https://doi.org/10.1016/j.wasman.2019.04.052

El-Sayed ESA, El-Sakhawy M, El-Sakhawy MA-M (2020) Non-wood fibers as raw material for pulp and paper industry. Nordic Pulp & Paper Research Journal 35:215–230. https://doi.org/10.1515/npprj-2019-0064

Furszyfer Del Rio DD, Sovacool BK, Griffiths S, et al (2022) Decarbonizing the pulp and paper industry: A critical and systematic review of sociotechnical developments and policy options. Renewable and Sustainable Energy Reviews 167:112706. https://doi.org/10.1016/j.rser.2022.112706

Ghose A, Chinga-Carrasco G (2013) Environmental aspects of Norwegian production of pulp fibres and printing paper. Journal of Cleaner Production 57:293–301. https://doi.org/10.1016/j.jclepro.2013.06.019

Kissinger M, Fix J, Rees WE (2007) Wood and non-wood pulp production: Comparative ecological footprinting on the Canadian prairies. Ecological Economics 62:552–558. https://doi.org/10.1016/j.ecolecon.2006.07.019

Lai C, Fan K, Cai Q, et al (2023) Low carbon and cost-effective pathways for specialty paper production in China. Sustainable Production and Consumption 39:556–568. https://doi.org/10.1016/j.spc.2023.05.033

Liu Z, Wang H, Hui L, et al (2018) Pulping and Papermaking of Non-Wood Fibers. In: Pulp and Paper Processing. IntechOpen

Pari L, Baraniecki P, Kaniewski R, Scarfone A (2015) Harvesting strategies of bast fiber crops in Europe and in China. Industrial Crops and Products 68:90–96. https://doi.org/10.1016/j.indcrop.2014.09.010

Ramdhonee A, Jeetah P (2017) Production of wrapping paper from banana fibres. Journal of Environmental Chemical Engineering 5:4298–4306. https://doi.org/10.1016/j.jece.2017.08.011

Sun M, Wang Y, Shi L (2018) Environmental performance of straw-based pulp making: A life cycle perspective. Science of The Total Environment 616–617:753–762. https://doi.org/10.1016/j.scitotenv.2017.10.250
